# Maternal cardiovascular-related single nucleotide polymorphisms, genes, and pathways associated with early-onset preeclampsia

**DOI:** 10.1371/journal.pone.0222672

**Published:** 2019-09-26

**Authors:** Paula Benny, Kelly Yamasato, Breck Yunits, Xun Zhu, Travers Ching, Lana X. Garmire, Marla J. Berry, Dena Towner

**Affiliations:** 1 University of Hawaii Cancer Center, Honolulu, Hawai’i, United States of America; 2 Department of Obstetrics, Gynecology, and Women’s Health, John A Burns School of Medicine, University of Hawaii at Manoa, Honolulu, Hawai’i, United States of America; 3 Department of Molecular Biosciences and Bioengineering, University of Hawaii at Manoa, Honolulu, Hawai’i, United States of America; 4 Department of Computational Medicine and Bioinformatics, University of Michigan, Ann Arbor, Michigan, United States of America; 5 Department of Cell and Molecular Biology, John A Burns School of Medicine, University of Hawaii at Manoa, Honolulu, Hawai’i, United States of America; University of Mississippi Medical Center, UNITED STATES

## Abstract

**Introduction:**

Preeclampsia is a medical condition complicated with hypertension and proteinuria during pregnancy. While preeclampsia affects approximately 5% of pregnancies, it remains without a cure. In addition, women who had preeclampsia during pregnancy have been reported to have an increased risk for cardiovascular disease later in life. However, the disease etiology and molecular mechanisms remain poorly understood. The paucity in the literature on preeclampsia associated maternal cardiovascular risk in different ethnic populations also present a need for more research. Therefore, the objective of this study was to identify cardiovascular/metabolic single nucleotide polymorphisms (SNPs), genes, and regulatory pathways associated with early-onset preeclampsia.

**Materials and methods:**

We compared maternal DNAs from 31 women with early-onset preeclampsia with those from a control group of 29 women without preeclampsia who delivered full-term normal birthweight infants. Women with multiple gestations and/or known medical disorders associated with preeclampsia (pregestational diabetes, chronic hypertension, renal disease, hyperthyroidism, and lupus) were excluded. The MetaboChip genotyping array with approximately 197,000 SNPs associated with metabolic and cardiovascular traits was used. Single nucleotide polymorphism analysis was performed using the SNPAssoc program in R. The Truncated Product Method was used to identify significantly associated genes. Ingenuity Pathway Analysis and Ingenuity Causal Network Analysis were used to identify significantly associated disease processes and regulatory gene networks respectively.

**Results:**

The early-onset preeclampsia group included 45% Filipino, 26% White, 16% other Asian, and 13% Native Hawaiian and other Pacific Islanders, which did not differ from the control group. There were no SNPs associated with early-onset preeclampsia after correction for multiple comparisons. However, through gene-based tests, 68 genes and 23 cardiovascular disease-related processes were found to be significantly associated. Associated gene regulatory networks involved cellular movement, cardiovascular disease, and inflammatory disease.

**Conclusions:**

Multiple cardiovascular genes and diseases demonstrate associations with early-onset preeclampsia. This unfolds new areas of research regarding the genetic determinants of early-onset preeclampsia and their relation to future cardiovascular disease.

## Introduction

Preeclampsia and eclampsia are global health conditions responsible for 10 to 15% of maternal deaths and up to 25% of stillbirths [1]. However, the pathophysiology of preeclampsia remains ill-defined [1, 2]. Investigation into the genetic determinants of preeclampsia may not only improve understanding of the underlying mechanisms of the disease, but could also lead to more effective interventions.

It is widely accepted that preeclampsia involves an underlying genetic predisposition, and multiple studies demonstrate a family history of preeclampsia to be a risk factor for the disease [3, 4]. Various inheritance models have been proposed, although the prevailing theory involves interactions between multiple susceptibility genes [4, 5]. Previous investigations into the genetic associations with preeclampsia have evaluated genes involved with processes disrupted in preeclampsia including thrombophilia, hemodynamics, endothelial function, cytokine signaling pathways, oxidative stress, lipid metabolism, endocrine function, and angiogenesis [4, 6]. A large proportion of these genetic association studies have examined single-nucleotide polymorphisms (SNPs) in a single gene [4], which are limited by the current knowledge of the pathogenesis of preeclampsia. In addition, genetic associations have been predominantly evaluated in genes with differential expression at disease manifestation, which may represent downstream effects rather than causal “predisposing” genes. Thus, the predisposing genetic differences may not be identified by this approach.

There are well-recognized associations between preeclampsia and cardiovascular and metabolic disease such as chronic hypertension and diabetes [2]. Moreover, women with a history of preeclampsia are more likely to subsequently develop these conditions [4]. The association between cardiovascular and metabolic disease and preeclampsia implies the presence of underlying genetic mechanisms common to the conditions, and elucidation of these mechanisms may improve the understanding of the pathogenesis of preeclampsia as well as future cardiovascular and metabolic disease risk assessment in women with a history of preeclampsia. In addition, there are racial differences in the rates of preeclampsia that persist after adjusting for other risk factors, with Native Hawaiian and other Pacific Islanders (NHOPI) and Filipinos demonstrating increased risk [7]. Thus, the genetics underlying preeclampsia may differ between racial groups. In addition, the paucity in the literature for NHOPI studies in preeclampsia and cardiovascular risk poses an unmet clinical need. Therefore, the objective of this study was to search for cardiovascular and metabolic disease-associated SNPs, genes, and gene regulatory networks in early-onset severe preeclampsia in a largely Asian and NHOPI cohort.

## Materials and methods

We conducted a case controlled study approved by the University of Hawai’i at Manoa institutional review board and conducted in accordance with the ethical standards outlined in the Declaration of Helsinki. Maternal DNA samples from maternal blood were obtained from the University of Hawaii Human Reproductive Biospecimen Repository. This repository is a tissue bank of over 9000 obstetric specimens paired with clinical data [8–10]. All patients included in the repository were recruited between 2005 and 2011 from Kapi‘olani Medical Center for Women and Children (Honolulu, Hawai‘i), a tertiary care center that performs approximately 6000 deliveries a year. Women were consented for inclusion in a biobank in which their samples would be used in approved studies, including those involving genetic information. Trained research personnel collected comprehensive clinical data at the time of delivery.

For this study we identified women with the diagnosis of early-onset preeclampsia delivered up through 34 weeks and 6 days gestational age. We chose a cutoff of 34 weeks and 6 days to account for minor errors in pregnancy dating and to include patients induced at 34 weeks 0 days gestation with delivery after 34 weeks 0 days. For the control group we selected age-matched and racially-matched subjects who delivered after 37 weeks 0 days without preeclampsia and had a neonate of normal birthweight. The inclusion criteria were (i) pregnant women above 18 years of age, (ii) preeclampsia diagnosis, and (iii) singleton pregnancy. The diagnosis of preeclampsia was made by the woman’s primary obstetrician. At the time of recruitment, the practice for diagnosis of preeclampsia necessitating delivery prior to 35 weeks included a diagnosis of preeclampsia (systolic blood pressure greater than or equal to 140 and/or diastolic blood pressure greater than or equal to 110 on 2 or more occasions at least 4 hours apart and 24 hour urine protein greater than or equal to 300 mg) and any of the following severe features: systolic blood pressure greater than or equal to 160 and/or diastolic blood pressure greater than or equal to 110 on 2 or more occasions at least 4 hours apart, thrombocytopenia (platelets less than 100,000), elevated liver enzymes, persistent right upper quadrant or epigastric pain, worsening renal insufficiency, pulmonary edema, new onset neurologic symptoms (eg: headache, vision changes), or intrauterine growth restriction. The exclusion criteria were patients with multiple gestations and/or medical disorders associated with preeclampsia, including pregestational diabetes, chronic hypertension, systemic lupus erythematosus, and renal disease to better isolate possible underlying genetic associations.

Genotyping was performed using the MetaboChip (Illumina, San Diego, CA), a genotyping array consisting of approximately 197,000 SNPs associated with metabolic and cardiovascular disorders. Loci included on the MetaboChip were selected from genome-wide association studies of 23 traits, including pregestational diabetes, myocardial infarction, coronary artery disease, hypertension, hyperlipidemia, and obesity. Additional SNPs identified through fine-mapping of 257 genetic loci associated with those traits are also included on the array [11].

We performed rigorous quality control metrics on our data (S1 Appendix). Statistical analysis was performed using the R SNPassoc package [12]. Gene-level analysis identified genes significantly associated with preeclampsia by taking into account the contributions from multiple SNPs within a gene. This was performed using the Truncated Product Method, which has been shown to have robust detection of associated genes in genome-wide association studies. for continuous variables [13]. Bonferroni corrections were used to adjust the gene level p-values. Principal component analysis, Manhattan plots and Phylogeny trees were carried out to determine case-control associations, SNP significance and ethnicity diversity respectively. After no significant SNPs were detected, we used pathway analysis using Ingenuity Pathway Analysis (IPA^**®**^, QIAGEN Redwood City) to group the genes that were significant at gene-level analysis based on biological functions or known disease pathogenesis. Ingenuity Causal Network Analysis (IPA^**®**^, QIAGEN Redwood City) was also used to create possible regulatory genetic networks using human-associated interactions. Clinical maternal data were analyzed via SPSS v. 22 (SPSS Inc. Chicago, IL), using Fisher’s exact test or chi-square test for categorical variables and the Mann-Whitney U test for continuous variables.

## Results

A total of 109 women were identified in the biorepository, of which 49 were excluded due to lack of adequate maternal sample, leaving 31 women with early-onset preeclampsia and 29 controls for the final analysis. Among women with early-onset preeclampsia, there were no statistical differences in race, maternal age (29.3 vs 29.8 years (P = 0.65)), body mass index (26.6 vs 30.1 kg/m^2^, (P = 0.05)), or gestational age at delivery (32.1 vs 32.8 weeks, (P = 0.35)) between those who were included and those excluded. Among control women, women who were excluded had a higher body mass index than those included (27.4 vs 20.8 kg/m^2^ (P = 0.014)), but race, maternal age, and gestational age at delivery were similar. Demographics of the early-onset preeclampsia and control groups are described in Table 1. There was no statistical difference in the racial composition between the two groups. Patients of Asian ancestry comprised 61.3% of the early-preeclampsia group and 51.7% of the control group. Of these subjects, Filipinos represented the largest Asian subgroup (45.2% and 44.8% for cases and controls, respectively). White patients represented the second most common racial group followed by Pacific Islanders.

**Table 1:** Subject demographics.

Demographic Variable	Early-Onset Preeclampsia (N = 31)	Control (N = 29)	P-value
Age [mean years, (SD)]	29.3 (6.0)	30.0 (5.5)	0.518
Gestational age at delivery [mean weeks, (SD)]	32.1 (2.2)	39.4 (0.8)	<0.001
Primigravida [N (%)]	12 (38.7)	9 (31.0)	0.595
Body mass index [mean kg/m^2^ (SD)]	26.6 (6.9)	20.8 (9.9)	0.009
Gestational diabetes [N (%)]	6 (19.4)	0 (0.0)	0.024
Male fetal sex [N (%)]	18 (58.1)	11 (37.9)	0.119
Birthweight [mean g [SD]]	1555 [439]	3332 [440]	<0.001
Race/Racial Subgroup [N (%)]			
Filipino	14 (45.2)	13 (44.8)	0.599
Other Asian	5 (16.1)	2 (6.9)	
White	8 (25.8)	11 (37.9)	
Pacific Islander	4 (12.9)	3 (10.3)	
≥ 1 Race/Racial Subgroup	13 (41.9)	10 (34.5)	0.603

There were no statistical differences between the two groups in maternal age and multiparity. However, the preeclamptic group had a higher mean body mass index (p = 0.009) and gestational diabetes rate (p = 0.024). Patients in the early-onset preeclampsia group delivered at a mean gestational age of 32 weeks compared to 39 weeks in the control group (p<0.001).

**Fig 1:**
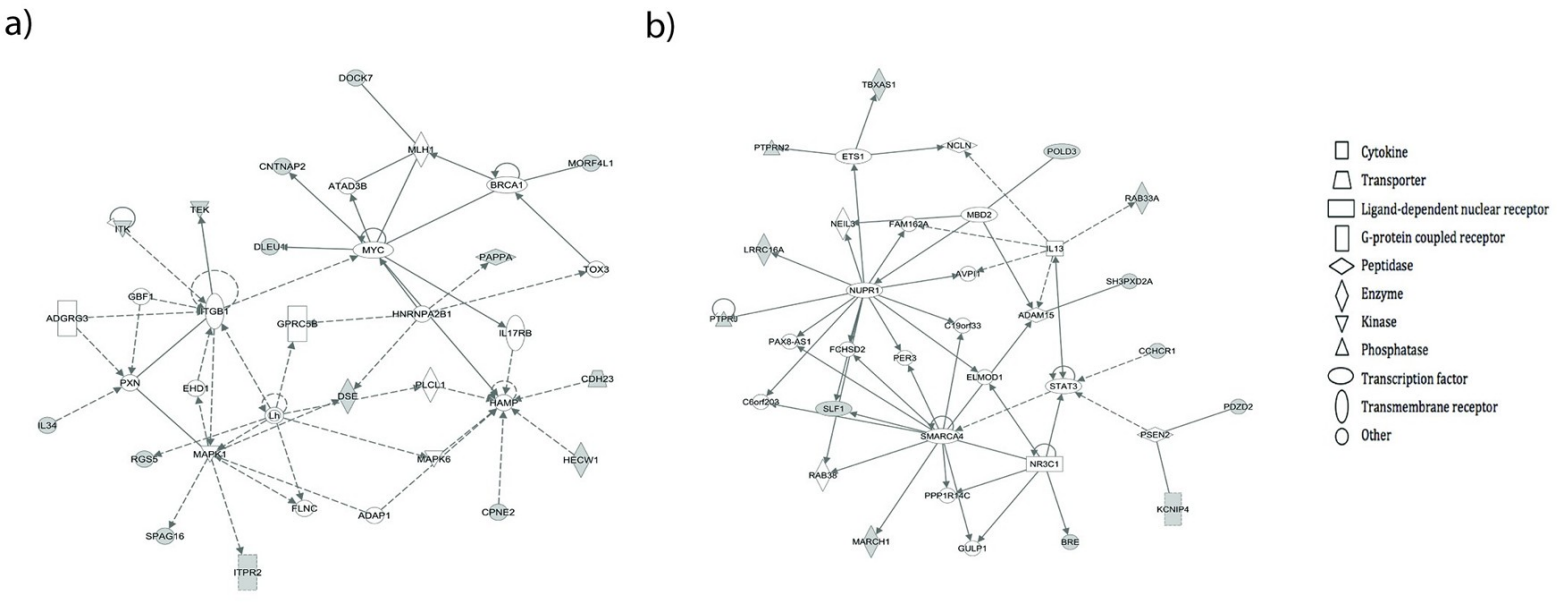
Gene regulatory networks associated with early-onset preeclampsia. Gene regulatory networks associated with early-onset preeclampsia related to a) cellular movement and b) cardiovascular disease. Solid lines represent direct associations and dotted lines represent indirect associations.

A total of 260 SNPs were associated with early-onset preeclampsia at a p value threshold of <0.001. Similar to other genome-wide association studies with small sample sizes, after correction for multiple comparisons none of these individual SNPs remained statistically significant. Rigorous quality control analysis confirmed this finding (S1 Appendix). However, at the gene level, 68 significantly associated genes were found. Table 2 represents a portion of these genes, which were selected based on clinical interest or involvement in disease pathways and regulatory networks. S2 Appendix describes the complete list of genes significantly associated with early-onset preeclampsia. Pathway analysis shows that these genes are involved in 23 cardiovascular-related diseases or biological processes (Table 3), and network analysis identified 8 related networks (Table 4 and Fig 1). The investigation into gene network pathways was to reveal groups of genes which were associated with preeclampsia and cardiovascular disease as these conditions are likely caused by and affect multiple genes. These networks are associated with diseases and functions such as cardiovascular and inflammatory disease, cellular movement, cell death and survival, and cardiovascular system development and function.

**Table 2:** Select genes significantly associated with early-onset preeclampsia.

Ensemble Gene ID	Gene Symbol	Adjusted P-value ^a^	Associated Functions/Diseases [14] (Partial List)
ENSG00000177000	*MTHFR*	8.11x10-4	Occlusive vascular disease
ENSG00000116641	*DOCK7*	4.06x10-5	Axon formation, Neuronal polarization
ENSG00000116473	*RAP1A*	4.06x10-5	Cell proliferation and adhesion
ENSG00000143248	*RGS5*	4.06x10-4	Hypertension, Endothelial apoptosis
ENSG00000092969	*TGFB2*	4.06x10-5	Cell proliferation, differentiation, adhesion, migration
ENSG00000158019	*BRE*	4.06x10-5	Leukemia, anti-apoptosis
ENSG00000145979	*TBC1D7*	5.68x10-3	Cellular growth and differentiation
ENSG00000111817	*DSE*	6.49x10-4	Tumor-rejection antigen
ENSG00000165029	*ABCA1*	4.14x10-3	Cellular lipid removal
ENSG00000149084	*HSD17B12*	4.06x10-5	Estrone to estradiol conversion in the ovary
ENSG00000077514	*POLD3*	2.56x10-2	DNA replication and repair
ENSG00000151702	*FLI1*	4.06x10-2	Ewing sarcoma, Leukemia
ENSG00000157368	*IL34*	4.06x10-5	Monocyte and macrophage differentiation and viability
ENSG00000178691	*SUZ12*	4.06x10-5	Endometrial stromal sarcoma
ENSG00000049759	*NEDD4L*	1.01x10-3	Hypertension, Epithelial sodium transport
ENSG00000087258	*GNAO1*	1.18x10-2	Early-onset epileptic encephalopathy

^a^ P-values obtained through the Truncated Product Method and corrected using the Bonferroni method

**Table 3:** Disease processes and functions associated with early-onset preeclampsia.

Disease/Function	Genes	P-value
Kawasaki’s Disease	*C3*, *CSMD1*, *DGKB*, *KCNP4*, *PDZD2*	3.48x10-7
Endothelial Tissue Permeability	*GNAO1*, *PTPRJ*, *TEK*	2.97x10-4
Loeys-Dietz Syndrome Type 4	*TGFB2*	4.35x10-3
Ghosal Hematodiaphyseal Dysplasia Syndrome	*TBXAS1*	4.35x10-3
Multiple Cutaneous and Mucosal Venous Malformation	*TEK*	4.35x10-3
Vascular Endothelial Permeability	*PTPRJ*, *TEK*	4.46x10-3
Acute Phase Atypical Hemolytic Uremic Syndrome	*C3*	8.69x10-3
Familial Defective Apo B-100	*APOB*	8.69x10-3
Endothelial Barrier Permeability	*GNAO1*	8.69x10-3
Microangiopathy	*C3*, *TEK*	1.25x10-2
Thrombus Formation	*C3*	1.30x10-2
Heterozygous Familial Hypercholesterolemia	*APOB*	1.30x10-2
Cavernous Hemangioma	*TEK*	1.73x10-2
Vascular Tumor	*BCL2*, *ROBO2*, *TEK*	1.76x10-2
Familial Vascular Disease	*TEK*, *TGFB2*	1.93x10-2
Familial Cardiovascular Disease	*APOB*, *TEK*, *TGFB2*	2.82x10-2
Fibromuscular Dysplasia	*TGFB2*	3.01x10-2

**Table 4:** Genetic regulatory networks associated with early-onset preeclampsia.

Molecules in Network	Score ^a^	Associated Diseases and Functions
ADAP1, ADGRG3, ATAD3B, BRCA1, **CDH23**, **CNTNAP2**, **CPNE2**, **DLEU1**, **DOCK7**, **DSE**, EHD1, FLNC, GBF1, GPRC5B, HAMP, **HECW1**, HNRNPA2B1, **IL34**, IL17RB, ITGB1, **ITK**, **ITPR2**, Lh, MAPK1, MAPK6, MLH1, **MORF4L1**, MYC, **PAPPA**, PLCL1, PXN, **RGS5**, **SPAG16**, **TEK**, TOX3	26	Cellular movement, Organismal development, Tissue morphology
ADAM15, AVPI1, **BRE**, C19orf33, C6orf203, **CCHCR1**, ELMOD1, ETS1, FAM162A, FCHHSD2, GULP1, IL13, **KCNIP4**, **LRRC16A**, **MARCH1**, MBD2, NCLN, NEIL3, NR3C1, NUPR1, PAX8-AS1, **PDZD2**, PER3, **POLD3**, PPP1R14C, PSEN2, **PTPRJ**, **PTPRN2**, RAB38, **RAB33A**, **SH3PXD2A**, **SLF1**, SMARCA4, STAT3, **TBXAS1**	22	Cardiovascular disease, Inflammatory disease, Organismal injury and abnormalities
**ANGPTL3**, **APOB**, **BCL2**, **C3**, CD6, CNKSR1, CYTIP, EGLN2, estrogen receptor, G0S2, Hsp70, IL36A, Jnk, **KIF26B**, **MAP3K3**, MAP3K10, MAP4K2, MED28, MICU1, **MMP24**, NFkB (complex), NOD1, NQO1, P38 MAPK, **PARK2**, **PRKCE**, RBM17, S100A12, TEAD, **TGFB2**, TRAF7, TRIO, UNC93B1, WNT4, **YAP1**	17	Cell death and survival, Skeletal and muscular disorders, Developmental disorders
ABCG5, AKR1C4, APOM, CAMK1D, **DGKB**, F11, FABP1, FTO, FUK, FUT3, FUT5, FUT6, FUT8, FUT10, FUT11, CGC, **GCNT3**, GIPR, **GLIS3**, GMDS, **GNAO1**, HHEX, HNF1A, HNF4A, INS, **JAZF1**, MTNR1B, NPC1L1, ONECUT1, PCSK1, **RORA**, RXRA, SOAT2, UGT1A7 (includes others), ZBED3	8	Post-translational modification, Carbohydrate metabolism, Molecular transport
MTF1, **TMC8**	2	Cell death and survival, Dermatological diseases and conditions, Embryonic development
PDX1, **SEZ6L**	2	Cellular development, Cellular growth and proliferation, Digestive system development and function
MGEA5, **TCF19**	2	Cardiovascular system development and function, Carbohydrate metabolism, Post-translational modification
**CLCA1**, MUC5AC, Mucin	2	Cell morphology, Digestive system development and function, Organ morphology

^a^ Score: Probability of finding *x* or more genes in a set of *n* randomly selected genes, where *x* = number of genes in the network significantly associated with preeclampsia and *n* = total number of genes in the network

Bolded genes represent those identified in the findings of this study

Also of note was the intergenic region of chromosome 5 between nucleotides 32879153–32903017 which showed the greatest significance on a Manhattan plot. Within this intergenic region 17 SNPs are associated with early-onset preeclampsia upon initial analysis, prior to the false discovery rate adjustment (S3 Appendix).

## Discussion

To our knowledge, this study is among the first to describe genetic associations in early-onset preeclampsia in a largely Asian and NHOPI population. In addition, while a history of preeclampsia is a well-established risk factor for future cardiovascular disease [15, 16], there is little information on genes common to both conditions. Some findings associated with preeclampsia in this study, such as MTHFR [17–19] and RGS5 [20] have been previously associated with preeclampsia, while maternal serum levels of TGFB2 [21] and ABCA1 [22] are altered in preeclampsia as well. While some effort has been undertaken to identify genetic associations between preeclampsia and cardiovascular disease, including PITX2 and chromosome locus 2q22, these studies were limited and used a candidate gene approach [23–25], as compared to our study which used a more global approach and studied under-represented populations such as the Native Hawaiians.

However, the majority of the associations we report are novel findings in comparison to PESNPdb, a comprehensive database of SNPs associated with preeclampsia [26], or other meta-analyses of preeclampsia-associated genes [27–29]. Specific to cardiovascular disease, a meta-analysis of datasets from microarray studies of preeclampsia and cardiovascular disease identified 22 differentially expressed genes common to both conditions [30]. Murphy et al. (2015) compared the proteomes of women with and without preeclampsia at six months postpartum and identified differentially-expressed peptides associated with cardiovascular disease in the preeclamptic group [31]. The genes identified in this study also differ from these previous reports. These differences may be related to the unique racial population and focus on early-onset preeclampsia as well as gene selection bias secondary to the use of the MetaboChip. In addition, we used maternal blood while many previous studies used placental samples. While placental genetic evaluation is highly relevant to preeclampsia, we chose maternal blood to elucidate the underlying maternal genetic predisposition. The availability of maternal blood compared to placental tissue may also make our findings more relevant to potential future clinical use. Although the unique cohort and sample selection likely contribute to our largely novel associations, these findings require further validation.

Some of the conditions and functions identified in our analysis, such as vascular endothelial permeability, have known roles in preeclampsia, supporting the validity of our findings. Fibromuscular dysplasia is another condition associated with an increased risk for preeclampsia [32]. In contrast, there is scant information on the relationship between Kawasaki Disease and preeclampsia, though strongly associated in our pathway analysis. Kawasaki disease is a vasculitis of unknown etiology in young children and is the most common cause of acquired heart disease in children in the developed world [33]. Further evaluation of the risk of preeclampsia in patients with a history of Kawasaki disease is warranted. Regulatory network analyses suggest processes involving cellular movement and proliferation, cardiovascular disease, and inflammatory disease to be associated with early-onset preeclampsia. Such associations are supported by the current understanding of preeclampsia. The understanding of not only the associated genes, but the regulation of these genes may be important for the development of potential pharmacogenomics therapies in the future.

This study focuses on early-onset preeclampsia, which is often considered to be a distinct entity from late-onset preeclampsia. Early-onset preeclampsia has a stronger familial component [34] and greater association with metabolic and cardiovascular conditions [35, 36] compared to late-onset disease. Differential genetic associations in early-onset and late-onset disease have been demonstrated in previous work [5,37]. However, many of the prior genome association studies either do not distinguish between the conditions or are limited to late-onset preeclampsia. Our findings therefore add more merit to the understanding of the genetic predisposition of early-onset preeclampsia exclusively, but may not apply to late-onset disease. As the aim of this work was to explore cardiometabolic genetic associations with early-onset preeclampsia, we chose to compare these women to a normotensive group to maximize identification of these potential variants.

The majority of prior work on the genetic associations with early-onset preeclampsia have involved White populations [4]. This study includes racial subgroups that have been relatively unstudied, including the Filipino and Pacific Islander populations that demonstrate an increased risk for gestational hypertension and preeclampsia [7,38]. Sun et al (2009) identified 72 genes with differential expression in six Chinese Han women with early-onset severe preeclampsia [39]. We did not find genetic associations with the genes identified in that study, perhaps due to our racially and clinically different population.

Though this study was inclusive of all eligible women regardless of race, the cohort remained limited in its sample size and stratification by racial group could not be performed. Though the biorepository contained over 9000 specimens, we ultimately identified only 109 eligible women due to our strict inclusion criteria. Excluding multiple medical comorbidities was important to isolating genetic predispositions, but markedly limited our sample size. Inadequate DNA, likely due to suboptimal collection or poor DNA quality, further limited our cohort. While the exclusion of samples with inadequate DNA is a potential source of bias, overall women with inadequate maternal sample were similar to those who were ultimately included.

In addition to racial heterogeneity, the racial composition of the case and control groups, while statistically the same, were not identical, with this difference potentially influencing our findings. Further study of larger, racially homogeneous groups are needed to confirm our findings. This work, however, provides possible directions for further research in this area and a primer for future related studies, especially candidate gene analysis. In addition, the implications of looking at gene pathway networks provides a more comprehensive understanding of the interaction between groups of genes and the underlying cellular processes associated with preeclampsia and cardiovascular disease. Finally, shifting definitions of preeclampsia can potentially introduce heterogeneity into cohorts studying this condition. However, in the time frame in which our samples were collected there were no major changes in recommendations regarding the diagnosis and management of preeclampsia.

Preeclampsia provides an opportunity for earlier recognition of a woman’s future cardiovascular health risk, yet this risk factor is often underappreciated. Future work may explore the relationships between preeclampsia, the genetic associations suggested by our results, and the development of clinical cardiovascular disease. Ultimately, the goal will be earlier recognition and improved preventative health care for women at increased risk for cardiovascular disease.

## Conclusions

Our findings suggest multiple cardiovascular-related genes and gene regulatory networks are associated with early-onset preeclampsia. This study builds upon the knowledge of the genetic contribution to early-onset preeclampsia and its relationship to cardiovascular disease in a relatively unstudied population. Such information may contribute to the understanding of the pathophysiology of preeclampsia as well as the development of pharmacogenomic treatments.

## Supporting information

S1 Appendix Quality control and data analysis. (DOC)

S2 Appendix Complete list of genes associated with early-onset preeclampsia. (DOC)

S3 Appendix Single nucleotide polymorphisms in a chromosome 5 intergenic region prior to adjustment for the false discovery rate. (DOC)
