# Supplementary material for: Maternal cardiovascular-related single nucleotide polymorphisms, genes, and pathways associated with early-onset preeclampsia
Source: PLoS One. 2019 Sep 26;14(9):e0222672. doi: 10.1371/journal.pone.0222672 (PMC6762142; doi:10.1371/journal.pone.0222672)
Supplement: S1 Appendix — (DOC) [file pone.0222672.s001.doc]

S1 Appendix: Quality control and data analysis


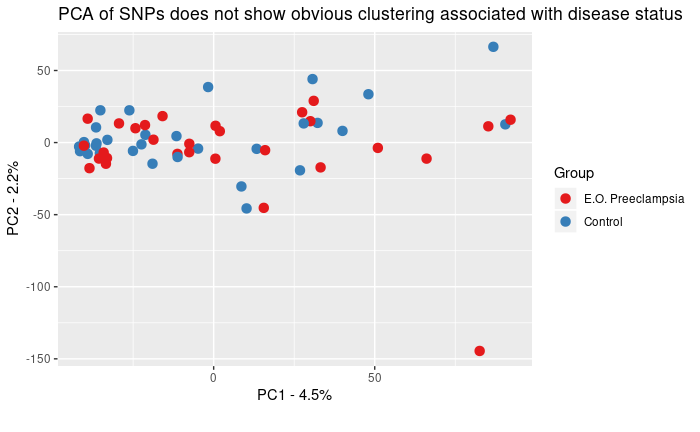


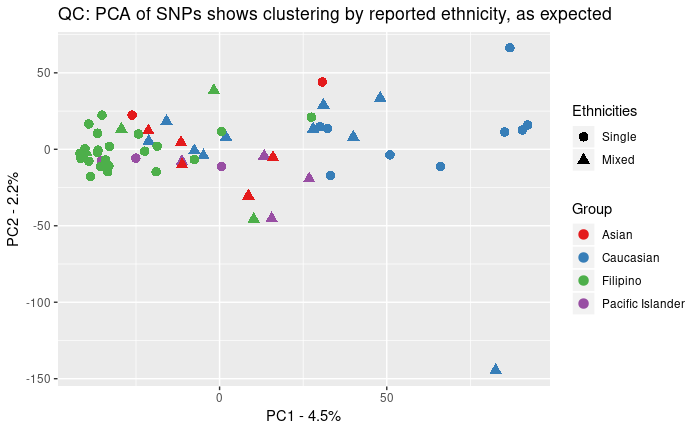


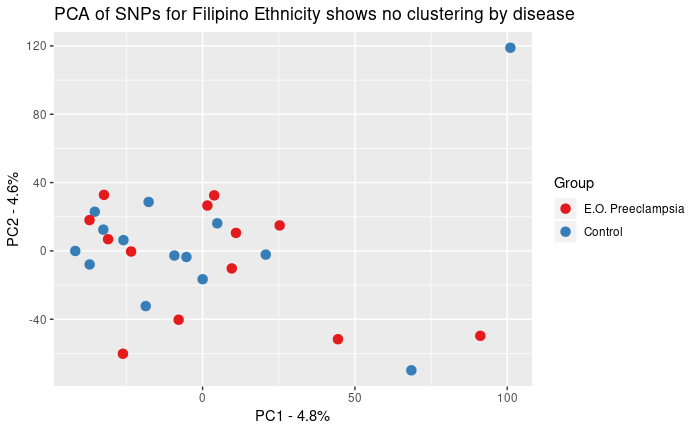


*
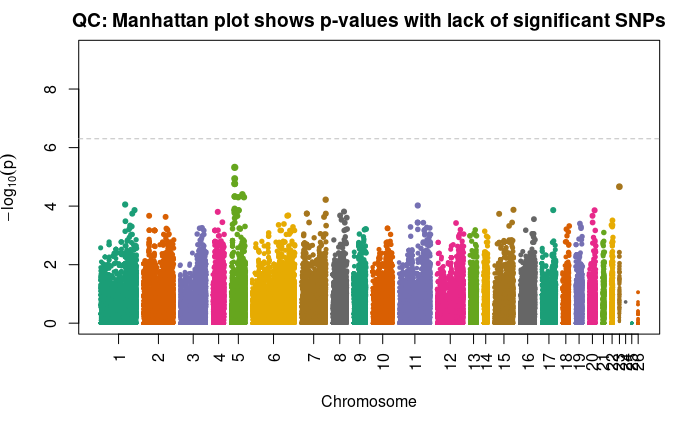
*


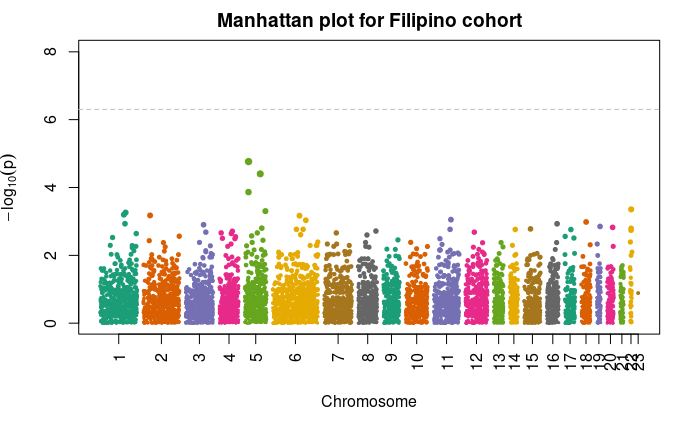


*
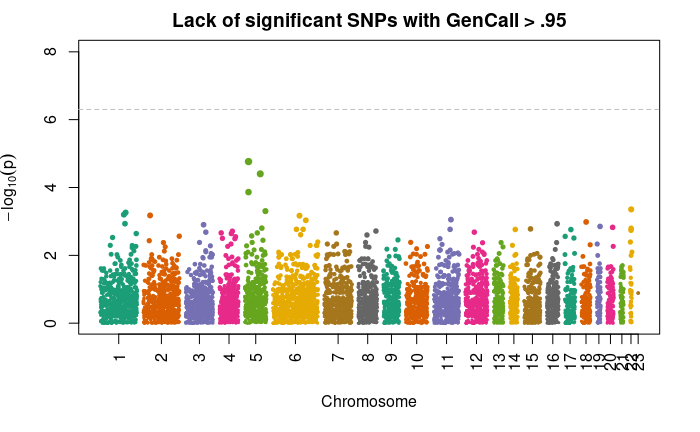
*


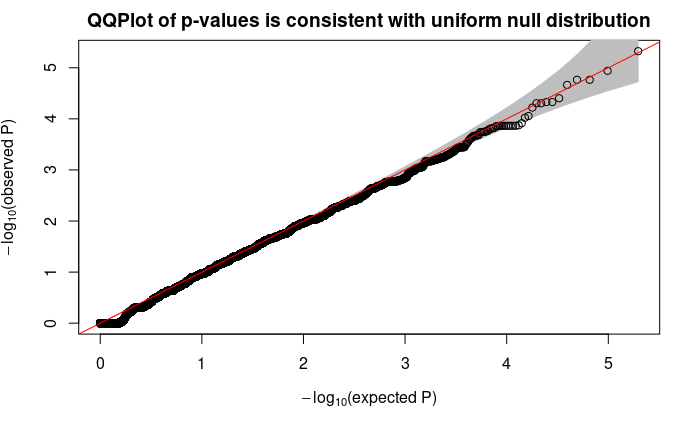


**Phylogeny tree after alignment demonstrates a rough concordance of ancestry with patient self-reported data**

*
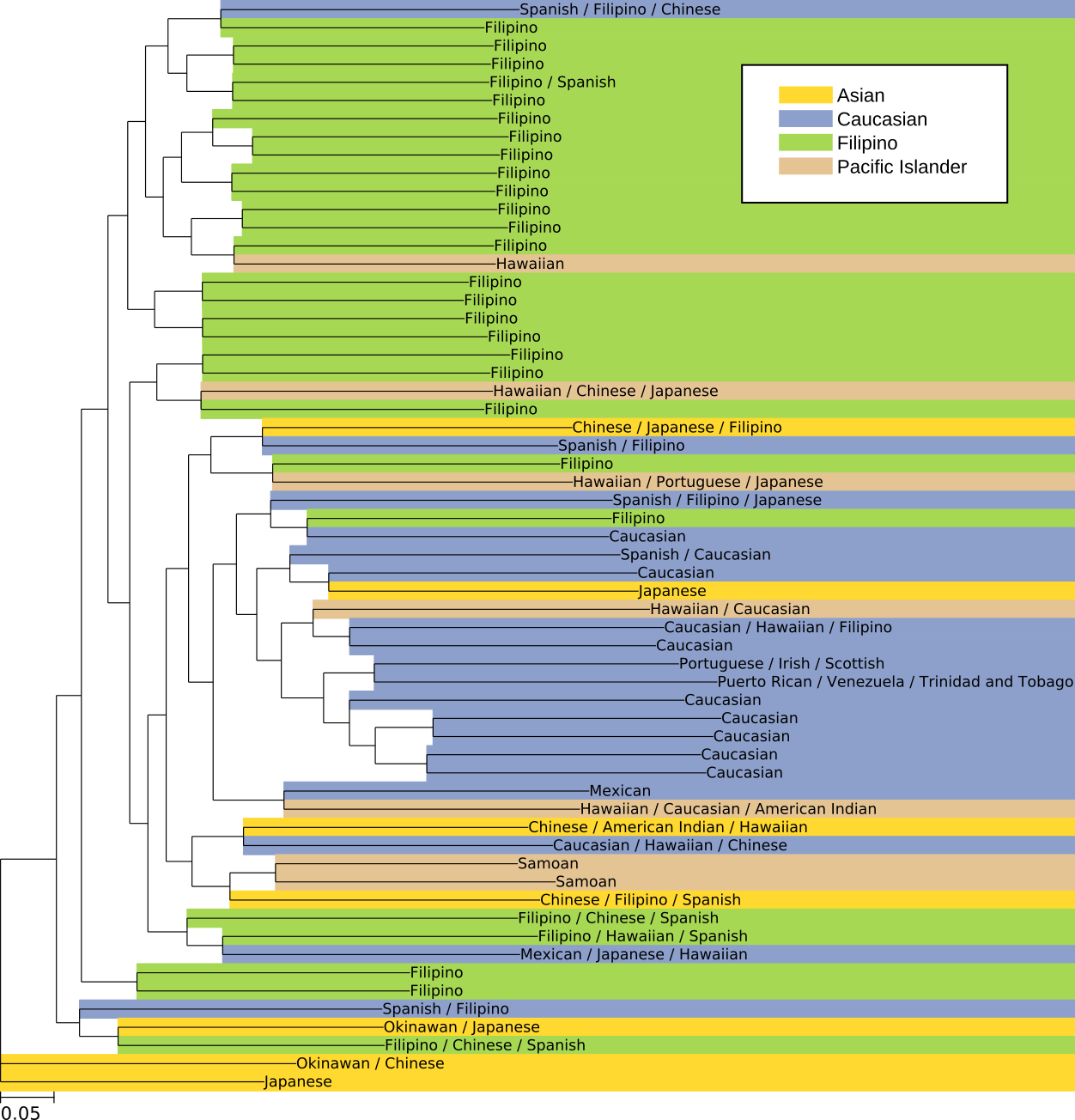
*
