# Supplementary material for: Maternal cardiovascular-related single nucleotide polymorphisms, genes, and pathways associated with early-onset preeclampsia
Source: PLoS One. 2019 Sep 26;14(9):e0222672. doi: 10.1371/journal.pone.0222672 (PMC6762142; doi:10.1371/journal.pone.0222672)
Supplement: S2 Appendix — (DOC) [file pone.0222672.s002.doc]

S2 Appendix: Complete list of genes associated with early-onset preeclampsia

| Chromosome Position | Ensemble  Gene ID | HGNC Symbol | Adjusted  P-value |
| --- | --- | --- | --- |
| 1.11845780-1.11866977 | ENSG00000177000 | *MTHFR* | 8.11x10^-4^ |
| 1.62920399-1.63153969 | ENSG00000116641 | *DOCK7* | 4.06x10^-5^ |
| 1.63106793-1.63107338 | ENSG00000213703 | None | 2.19x10^-2^ |
| 1.112084840-1.112259313 | ENSG00000116473 | *RAP1A* | 4.06x10^-5^ |
| 1.160295894-1.160296006 | ENSG00000202078 | None | 8.93x10^-4^ |
| 1.163080911-1.163291577 | ENSG00000143248 | *RGS5* | 4.06x10^-4^ |
| 1.163131465-1.163182813 | ENSG00000232892 | None | 2.19x10^-2^ |
| 1.218519577-1.218617961 | ENSG00000092969 | *TGFB2* | 4.06x10^-5^ |
| 1.228270361-1.228286912 | ENSG00000143761 | *ARF1* | 1.26x10^-3^ |
| 1.245318287-1.245872733 | ENSG00000162849 | *KIF26B* | 4.79x10^-2^ |
| 2.27994584-2.28210954 | ENSG00000243147 | *MRPL33* | 4.06x10^-5^ |
| 2.28112808-2.28561768 | ENSG00000158019 | *BRE* | 4.06x10^-5^ |
| 2.56093102-2.56151274 | ENSG00000115380 | *EFEMP1* | 4.46x10^-2^ |
| 2.182818968-2.182996125 | ENSG00000150722 | *PPP1R1C* | 4.06x10^-5^ |
| 3.155755490-3.156256545 | ENSG00000169282 | *KCNAB1* | 7.71x10^-4^ |
| 4.81256874-4.81884910 | ENSG00000197826 | *C4orf22* | 4.06x10^-5^ |
| 5.33523640-5.33892297 | ENSG00000151388 | *ADAMTS12* | 1.83x10^-2^ |
| 6.157652360-6.157661384 | ENSG00000253449 | None | 4.06x10^-5^ |
| 6.13266774-6.13328815 | ENSG00000145979 | *TBC1D7* | 5.68x10^-3^ |
| 6.25279306-6.25620758 | ENSG00000079691 | *LRRC16A* | 4.06x10^-5^ |
| 6.31110216-6.31126015 | ENSG00000204536 | *CCHCR1* | 4.06x10^-5^ |
| 6.31126319-6.31134936 | ENSG00000137310 | *TCF19* | 5.28x10^-3^ |
| 6.31261685-6.31269419 | ENSG00000256166 | None | 4.06x10^-5^ |
| 6.31830969-6.31846823 | ENSG00000204385 | *SLC44A4* | 2.92x10^-3^ |
| 6.109487036-6.109592217 | ENSG00000243587 | *C6orf183* | 4.06x10^-5^ |
| 6.116575336-6.116762424 | ENSG00000111817 | *DSE* | 6.49x10^-4^ |
| 7.27870192-7.28220362 | ENSG00000153814 | *JAZF1* | 1.50x10^-3^ |
| 9.3824127-9.4348392 | ENSG00000107249 | *GLIS3* | 4.06x10^-5^ |
| 9.78505560-9.78977255 | ENSG00000099139 | *PCSK5* | 4.06x10^-5^ |
| 9.107543283-9.107690518 | ENSG00000165029 | *ABCA1* | 4.14x10^-3^ |
| 10.104238986-10.104262482 | ENSG00000138107 | *ACTR1A* | 5.28x10^-3^ |
| 11.105348285-11.105615301 | ENSG00000107957 | *SH3PXD2A* | 1.79x10^-2^ |
| 11.2465914-11.2870339 | ENSG00000053918 | *KCNQ1* | 2.96x10^-3^ |
| 11.2629558-11.2721224 | ENSG00000269821 | *KCNQ1OT1* | 4.06x10^-5^ |
| 11.27528385-11.27719721 | ENSG00000245573 | *BDNF-AS* | 4.06x10^-5^ |
| 11.43577986-11.43878167 | ENSG00000149084 | *HSD17B12* | 4.06x10^-5^ |
| 11.45868669-11.45904798 | ENSG00000121671 | *CRY2* | 4.06x10^-5^ |
| 11.47738072-11.47788995 | ENSG00000109920 | *FNBP4* | 4.06x10^-5^ |
| 11.74204896-11.74380162 | ENSG00000077514 | *POLD3* | 2.56x10^-2^ |
| 11.128556430-11.128683162 | ENSG00000151702 | *FLI1* | 4.06x10^-2^ |
| 12.26274924-12.26452223 | ENSG00000123096 | *SSPN* | 4.06x10^-5^ |
| 12.26364097-12.26488789 | ENSG00000256234 | None | 4.46x10^-2^ |
| 12.45875654-12.46004403 | ENSG00000257657 | None | 4.06x10^-5^ |
| 12.88442793-12.88535993 | ENSG00000198707 | *CEP290* | 4.06x10^-4^ |
| 12.93710911-12.93771512 | ENSG00000257252 | None | 2.27x10^-2^ |
| 12.109785708-12.109893328 | ENSG00000174527 | *MYO1H* | 2.15x10^-3^ |
| 12.109883215-12.109885302 | ENSG00000255655 | None | 5.28x10^-3^ |
| 12.121416346-12.121440315 | ENSG00000135100 | *HNF1A* | 4.46x10^-2^ |
| 13.109248500-13.109860355 | ENSG00000041515 | *MYO16* | 2.96x10^-2^ |
| 15.72968124-15.72978490 | ENSG00000175202 | *HIGD2B* | 1.99x10^-2^ |
| 15.72978527-15.73030817 | ENSG00000140463 | *BBS4* | 4.06x10^-5^ |
| 15.76640526-15.77197785 | ENSG00000140386 | *SCAPER* | 4.06x10^-5^ |
| 16.19727778-16.19868907 | ENSG00000174628 | *IQCK* | 1.30x10^-2^ |
| 16.52586002-16.52686017 | ENSG00000249231 | *CASC16* | 4.06x10^-5^ |
| 16.56225302-16.56391356 | ENSG00000087258 | *GNAO1* | 1.18x10^-2^ |
| 16.70514471-16.70557468 | ENSG00000103051 | *COG4* | 3.00x10^-2^ |
| 16.70557691-16.70608820 | ENSG00000189091 | *SF3B3* | 4.06x10^-5^ |
| 16.70613798-16.70694585 | ENSG00000157368 | *IL34* | 4.06x10^-5^ |
| 17.30187923-17.30228784 | ENSG00000108651 | *UTP6* | 6.49x10^-3^ |
| 17.30264037-17.30328064 | ENSG00000178691 | *SUZ12* | 4.06x10^-5^ |
| 18.55711599-18.56068772 | ENSG00000049759 | *NEDD4L* | 1.01x10^-3^ |
| 19.65183783-19.65566856 | ENSG00000265533 | None | 4.14x10^-2^ |
| 19.19322782-19.19363042 | ENSG00000130287 | *NCAN* | 8.12x10^-3^ |
| 19.45453301-19.45457264 | ENSG00000267114 | None | 4.99x10^-2^ |
| 19.51014857-19.51017947 | ENSG00000204653 | *ASPDH* | 2.64x10^-2^ |
| 19.51020149-19.51071302 | ENSG00000131409 | *LRRC4B* | 4.06x10^-5^ |
| 19.51058627-19.51061226 | ENSG00000268231 | None | 8.93x10^-4^ |
| 22.33558212-22.34318829 | ENSG00000133424 | *LARGE* | 3.65x10^-2^ |
