# Supplementary material for: Maternal cardiovascular-related single nucleotide polymorphisms, genes, and pathways associated with early-onset preeclampsia
Source: PLoS One. 2019 Sep 26;14(9):e0222672. doi: 10.1371/journal.pone.0222672 (PMC6762142; doi:10.1371/journal.pone.0222672)
Supplement: S3 Appendix — (DOC) [file pone.0222672.s003.doc]

S3 Appendix: Single Nucleotide Polymorphisms in a Chromosome 5 Intergenic Region Prior to Adjustment for the False Discovery Rate

| Single Nucleotide Polymorphism | P-value * |
| --- | --- |
| chr5.32879153 | 0.000247644 |
| chr5.32879250 | 0.000343399 |
| chr5.32879340 | 0.000343399 |
| chr5.32882780 | 0.000288758 |
| chr5.32884755 | 0.000136686 |
| chr5.32888767 | 1.15E-05 |
| chr5.32889877 | 4.95E-05 |
| chr5.32890721 | 0.000136686 |
| chr5.32891446 | 0.000136686 |
| chr5.32891665 | 0.000257502 |
| chr5.32896489 | 0.000136686 |
| chr5.32897461 | 1.72E-05 |
| chr5.32898472 | 4.74E-06 |
| chr5.32900830 | 0.000362376 |
| chr5.32901792 | 0.000136686 |
| chr5.32901811 | 1.72E-05 |
| chr5.32903017 | 4.95E-05 |

* P-value *prior to* adjustment for the false discovery rate. Following adjustment no single P-value was statistically significant
